# Gallium Nitride High Electron Mobility Transistor Device with Integrated On-Chip Array Junction Temperature Monitoring Unit

**DOI:** 10.3390/mi16030304

**Published:** 2025-03-04

**Authors:** Yukuan Chang, Yue Su, Mingke Xiao, Jiatao Wu, Xu Zhang, Hongda Chen

**Affiliations:** 1 School of Intelligent Science and Technology, Hangzhou Institute for Advanced Study, University of Chinese Academy of Sciences, Hangzhou 310024, China; changyukuan@ucas.ac.cn (Y.C.); xiaomingke23@mails.ucas.ac.cn (M.X.); wujiatao23@mails.ucas.ac.cn (J.W.); hdchen@ucas.ac.cn (H.C.); 2 Institute of Semiconductors, Chinese Academy of Sciences, Beijing 100864, China; zhangxu@semi.ac.cn

**Keywords:** GaN HEMT, junction temperature, temperature monitoring unit, thermal distribution, monitoring accuracy

## Abstract

Herein, we present a novel method for junction temperature monitoring of GaN HEMT devices to achieve real-time temperature perception at different locations on the device surface. Through sputtering patterned Ti/Pt thermistor strips on the surface of a GaN HEMT device to construct an on-chip array junction temperature monitoring unit, the thermal distribution of the device during operation is fully reflected. The developed temperature monitoring unit exhibited a desirable temperature coefficient of resistance of 0.183%/°C in the range of 25 °C to 205 °C. Comparison with the thermal imager shows that the integrated temperature monitoring unit can accurately reflect the real-time temperature with a monitoring accuracy of more than 95%, which helps to improve the long-term reliability of GaN power devices under actual application conditions of high frequency and high power density.

## 1. Introduction

In recent years, GaN devices have attracted widespread attention in the field of power electronics due to their excellent electrical properties and high efficiency. Compared with traditional silicon-based devices, GaN power devices have the characteristics of low on-resistance, high voltage resistance and high switching frequency, which are particularly suitable for high-frequency, cost-sensitive, and moderate-power application scenarios, such as photovoltaics and energy storage, consumer electronics, electric vehicles, data centers, etc. [1,2]. However, as GaN-based devices move towards higher power density and smaller size, more heat is generated in a more confined area, and how to effectively manage the heat generated by the device becomes a pressing issue. For GaN HEMT devices, heat is mainly generated by the interaction between electrons and phonons in the two-dimensional electron gas (2DEG) region. This accumulation of heat power consumption leads to an increase in the junction temperature of the chip, which will bring serious reliability problems such as dynamic on-resistance degradation, threshold voltage drift, current collapse, etc. Therefore, how to measure the device temperature sensitively and accurately so as to effectively monitor the device status and provide over-temperature protection is a challenge in the current industry research and exploration.

Accurately evaluating the junction temperature of power devices is the key basis for power device life prediction, thermal management and reliability research, and has extremely important practical and economic significance [3,4,5]. To monitor the junction temperature of power devices, four main types of measurement methods have been proposed, including the optical method [6,7,8,9], thermal resistance model prediction method [10], temperature-sensitive electrical parameter method [11,12,13,14,15,16] and physical measurement method [17]. Optical methods such as infrared thermography and Raman spectroscopy enable the accurate evaluation of the temperature distribution across the GaN devices [6,7]. Choi et al. proposed that the use of frequency shifts in both the E2 (high) and A1 (LO) phonon modes offers accurate and time-efficient means to determine the state of temperature and thermal stress in operating AlGaN/GaN HEMTs [8]. However, these optical methods require the device to be opened and debonded, which is invasive. The thermal impedance model prediction method calculates the junction temperature of the device based on the actual power dissipation of the device under test and the thermal network model [10]. Although this type of method is non-invasive, it requires an accurate multi-order thermal network model and real-time updating of model node parameters, which has high computational costs. The temperature-sensitive electrical parameter method indirectly estimates the junction temperature by using some electrical parameters that are closely related to the junction temperature of the power device [11]. For depletion-mode GaN HEMT devices, the forward conduction voltage drop of the gate Schottky junction is usually used as a temperature-sensitive parameter [12,13]. For enhancement-mode GaN HEMT devices, due to the poor linearity of the gate voltage with temperature, the on-resistance of the device is usually used as a temperature-sensitive parameter to measure the device channel temperature and thermal resistance [14]. Common physical contact temperature measurement methods include the thermistor method and thermocouple temperature measurement method. Reiner et al. reported a method to monitor the internal temperature of the device by using a thin film metal as the temperature sensor on a high-voltage GaN-based power HEMT [17]. However, the design flaw of this device is that the temperature detection unit is placed between different cells of the device, rather than at the main heating point inside the device, which will cause the detection temperature to be much lower than the highest junction temperature inside the device. At present, most of the junction temperature measurement methods of GaN power devices have one or some of the following defects such as damage to the device structure, low junction temperature test accuracy, and poor compatibility and versatility with different device structures and materials.

In this paper, we demonstrate a new structure of GaN power devices with on-chip integrated distributed junction temperature monitoring units, which enables the real-time monitoring of the temperature at multiple points on the device surface by sputtering and patterning Ti/Pt thermistor strips on the device surface. Through real-time monitoring of the junction temperature, the device can be ensured to operate within the safe temperature range and prevent performance degradation or failure caused by overheating. The integrated temperature monitoring unit exhibits high sensitivity, achieving a temperature coefficient of resistance (TCR) of 0.183%/°C in the range of 25 °C to 205 °C. The experimental results show that this monitoring unit can accurately reflect the thermal distribution of the device in real time under working conditions, which is highly consistent with the test results of the thermal imager, with an accuracy rate of more than 95%. This low-cost method helps to improve the long-term reliability and performance stability of GaN power devices in high-frequency and high-power-density applications.

## 2. Experimental Details

**Figure 1:**
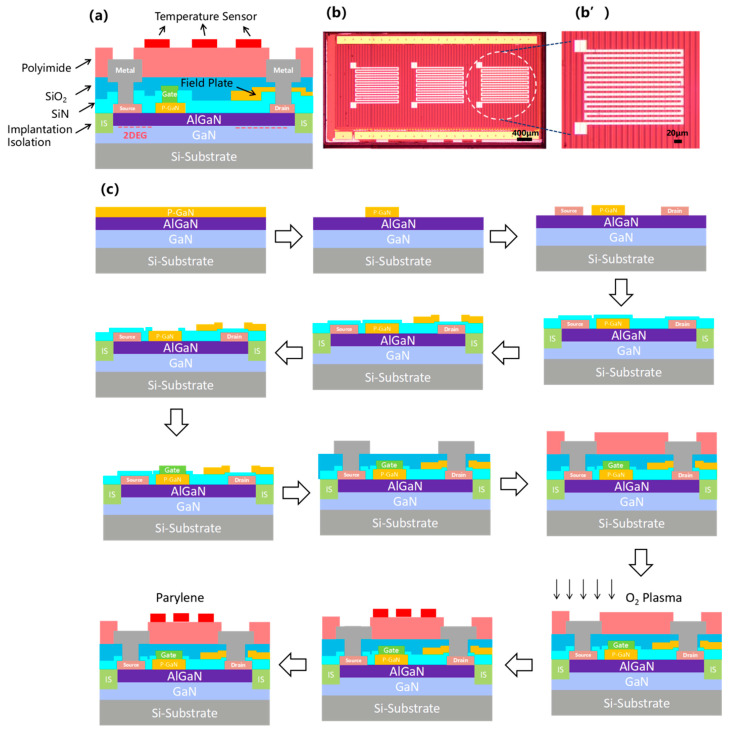
(**a**) The cross-sectional structure of GaN-HEMT power device cell with integrated temperature monitoring unit. (**b**) Photograph of the GaN power device with an embedded temperature sensing unit taken by a digital camera. (**b’**) A magnified view of the temperature sensing unit. (**c**) Schematic of the preparation procedure for GaN power devices with integrated distributed junction temperature monitoring unit. (Arrows denote the sequential direction of the fabrication steps).

The schematic diagram of the cell structure of a p-GaN gate GaN device with an integrated distributed temperature monitoring unit is illustrated in Figure 1a. It can be found from Figure 1a that the spatial position of the on-chip temperature monitoring unit is close to the polyimide (PI) passivation layer in the vertical direction.

The fabrication of the GaN power devices with an integrated distributed junction temperature monitoring unit was carried out with the procedures illustrated in Figure 1c. The process began with alignment and marking of the p-GaN layer on the silicon-based GaN epitaxial wafer. This was followed by exposure, photolithography, and dry etching to remove the p-GaN cap layer. Next, a Ti/Al/Ni/Au multilayer alloy with a low work function was deposited at the source and drain positions, and then a rapid annealing was performed at 830 °C for 60 s to form a stable ohmic contact between the Ti/Al/Ni/Au alloy and the AlGaN layer [18]. After the above steps were completed, ion implantation was performed in the active area of the device to achieve effective isolation between different devices. Subsequently, a layer of SiN_X_ was deposited as a passivation layer to protect the device from damage in subsequent processes. Next, SiN_X_ material was deposited to form a field plate structure to adjust the channel electric field distribution, thereby increasing the breakdown voltage of the device. Next, a gate metal hole was drilled above the P-GaN cap layer and a Ni/Au alloy layer was deposited at the location of the hole, followed by rapid annealing at 400 °C in a pure nitrogen atmosphere for 10 min to form a Schottky contact [19,20]. Based on the above steps, the SiO_2_ passivation layer was deposited. Etching was performed on the source and drain electrodes, and the metal electrodes were led out to form the electrode PAD. Afterwards, a polyimide top passivation layer was spin-coated and treated with oxygen plasma to increase surface adhesion. A 20 nm-thick titanium (Ti) film and a 100 nm-thick platinum (Pt) film were sputtered sequentially on the surface of the PI film to prepare the temperature sensing unit. In order to protect the prepared Ti/Pt thermistor strip, a 500 nm-thick parylene polymer film was vapor-deposited on the surface of the device. After that, the sample was placed on a hot plate, quickly heated to 120 °C, and naturally cooled to room temperature to improve the stability of the metal sensitive material. Figure 1b exhibits the photograph of the fabricated GaN power device with an integrated temperature sensing unit taken by a digital camera (The total gate length is 15 mm). In the inset (b’) of Figure 1b, we also show a magnified view of an individual temperature sensing unit, which is highlighted by a dashed white line. From the magnified figure, it can be found that the shape of the platinum resistance strip is designed as a grating structure to facilitate measurement, and the spacing between the platinum resistance strips is set to 20 μm.

Typical electrical performance of power devices was tested using Agilent B1505A semiconductor characterization analyzer (Keysight Technologies, Santa Rosa, CA, USA), including transfer and output characteristics. The sensing performance of the temperature monitoring unit was measured with a homemade testing machine, which is composed of a ceramic heating plate (Harry Gestigkeit PZ44, Harry Gestigkeit GmbH, Hamburg, Germany) and a source meter (Keithley 2450, Tektronix, Cleveland, OH, USA). The thermal characteristics of power devices were characterized by infrared thermal imager (FOTRIC 220s, FOTRIC Technologies, Shanghai, China).

## 3. Results and Discussion

**Figure 2:**
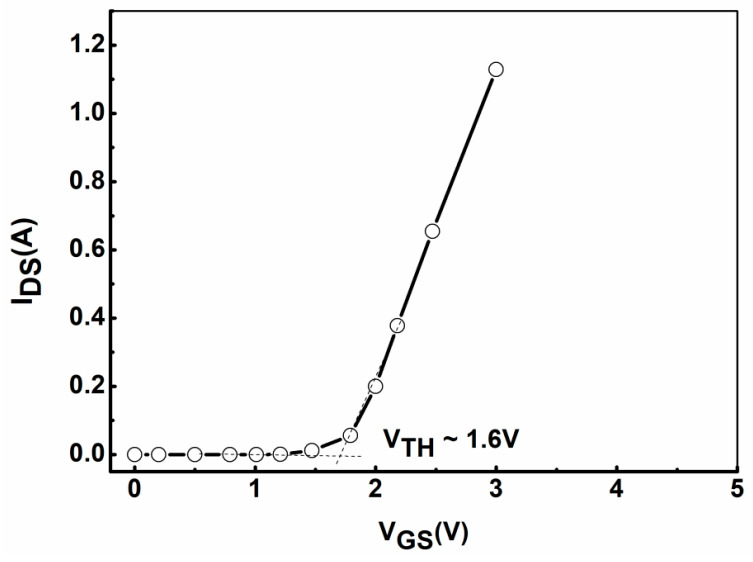
Transfer characteristics of the fabricated GaN-HEMT at V_DS_ = 3 V.

**Figure 3:**
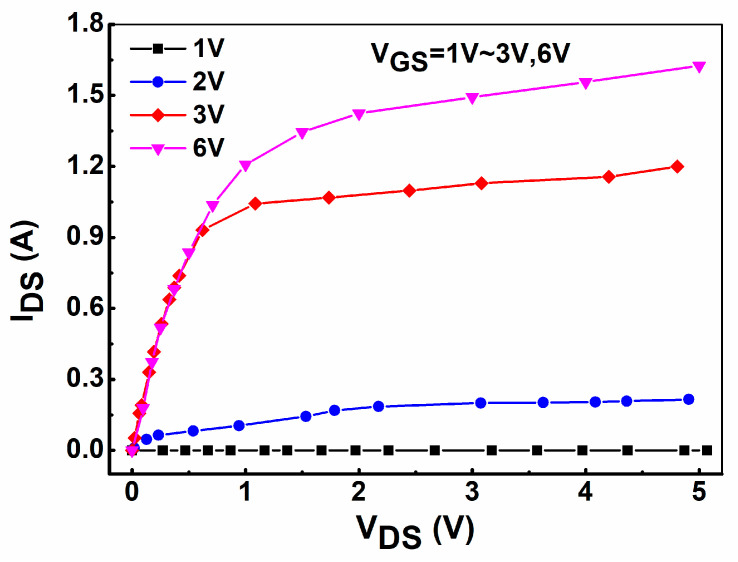
Output characteristics of the fabricated GaN-HEMT.

After the preparation of the GaN HEMT device with integrated on-chip distributed temperature monitoring unit was completed, the basic electrical performance test of the device was carried out, including transfer characteristic and output characteristic curve tests. In addition, the resistance value test and temperature coefficient characteristic test of the integrated temperature monitoring unit were also carried out. Figure 2 shows the transfer characteristic test curve of the fabricated GaN-HEMT device. During the transfer characteristic test of the sample, the drain voltage was set to 3 V and the gate voltage scanning range was set to 0 V to 3 V. As can be seen from Figure 2, the device normally operates in an off mode, achieving a threshold voltage V_TH_ as high as 1.6 V (defined by linear extrapolation [21]). The output characteristic curve of AlGaN/GaN HEMT devices is the key to judging the effective output power of power devices. Figure 3 shows the output characteristic curve of the GaN HEMT device. The gate bias voltage of the device increases from 1 V to 6 V. As can be seen from Figure 3, the maximum saturation current of the prepared device is 1.5 A when the gate voltage is 6 V. Based on the above transfer and output characteristic tests and analysis, the basic performance of GaN power devices is normal, indicating that the internal integrated temperature monitoring unit has no effect on the basic electrical characteristics of the device.

**Figure 4:**
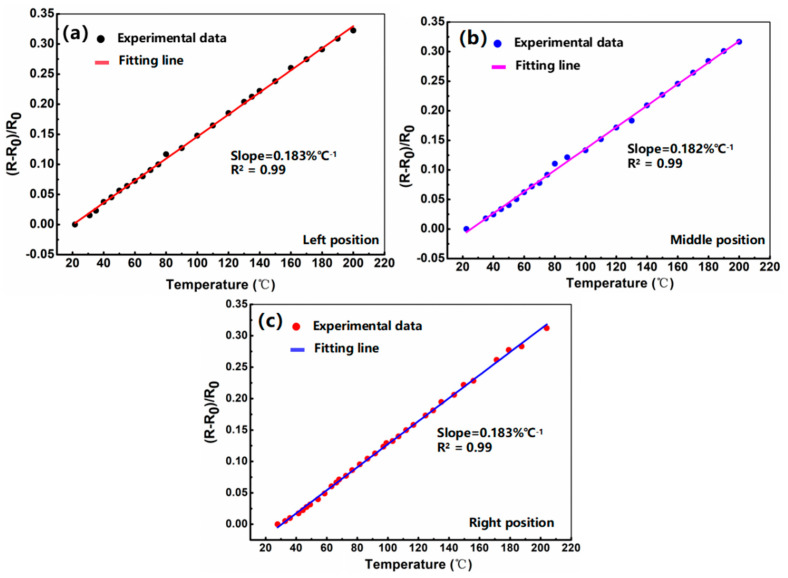
Relative resistance changes versus the applied temperature of the sensing unit at different positions, corresponding to the left (**a**), middle (**b**), and right (**c**) positions, respectively.

Next, we tested and analyzed the performance of the temperature monitoring unit embedded in the device. Herein, no voltage was applied to the GaN device, and only the two electrodes (PAD) of the distributed temperature monitoring unit were connected to an external power supply to measure the temperature coefficient. In order to measure the sensors’ response to temperature variations, a ceramic heating plate was used to simulate a specific thermal environment, with a source meter to detect resistance changes in real time. The test temperature was set from 25 °C to 205 °C. After reaching a given temperature, it is maintained for 5 min to facilitate measurement. In Figure 4a–c, we show the relationship between resistance response versus the temperature loading of the sensing unit at different positions, corresponding to the left (Figure 4a), middle (Figure 4b), and right (Figure 4c), positions, respectively. The obtained data show that the fabricated temperature sensing devices exhibit excellent linearity (R^2^ = 0.99). The temperature coefficient of resistance, as a key parameter of the temperature sensor, can be defined as TCR = (∆R/R_0_)/∆T, where ∆R denotes a change in resistance before and after a certain temperature is applied, R_0_ denotes the pristine resistance value, and ∆T represents the change in the applied temperature. The maximum fitted TCR of the temperature sensor is 0.183%°C^−1^ for the range of 25 °C to 205 °C, which is comparable to that reported in the previous literature [22,23]. It can also be observed that the resistance temperature coefficient values of the temperature sensors at different positions are all in the range of 0.182–0.183%°C^−1^, indicating that the prepared temperature sensing unit exhibits excellent consistency.

**Figure 5:**
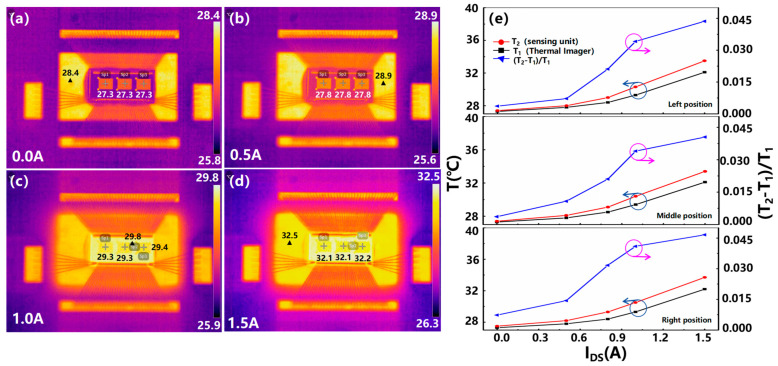
(**a**–**d**) Thermal images of the GaN-HEMT power device with integrated temperature monitoring unit under different drain current conditions, corresponding to constant currents of 0.0 A (**a**), 0.5 A (**b**), 1.0 A (**c**), and 1.5 A (**d**). (**e**) Comparison of the temperatures measured by the infrared imager and the temperature monitoring units at different locations under normal working conditions of the device. (Arrows associate the data curves with their vertical axes).

Furthermore, we investigated the accuracy of the temperature sensing unit for temperature monitoring when the power device actually generates heat. First, a 3 V voltage was applied to the gate of the device under test and a constant current was passed through the drain of the device to make the GaN HEMT device work normally and produce a self-heating effect. The temperature sensing unit was used to monitor the corresponding resistance value in real time, and the corresponding temperature can be calculated through the functional relationship. Herein, it should be noted that when the internal temperature monitoring unit of the device operates with an external voltage, the basic electrical characteristics of the GaN power device are not affected. In addition, an infrared imager was used to quickly obtain the accurate temperature value of the device. Herein, the infrared thermal imager was calibrated with the heating instrument in advance to ensure the accuracy of the measurement data. By comparing the measured data, the function and accuracy of the temperature monitoring unit can be determined. Figure 5a–d exhibit the thermal images of the GaN-HEMT power device with an integrated temperature monitoring unit under different drain current conditions (chip-on-board packaging technology). As can be seen from Figure 5a–d, as the drain current increases from 0.0 A to 1.5 A, the heating of the GaN HEMT device gradually increases, which is mainly attributed to the combined effects of resistance loss, carrier scattering, parasitic effects, and nonlinear characteristics. A comparison of the temperatures measured by the infrared thermal imager and the temperature monitoring units at different positions is demonstrated in Figure 5e. It can be observed that T_1_ calculated by the function relationship is greater than the value of T_2_ as measured by the infrared imager, and the absolute difference between T_1_ and T_2_ increases with the increase in drain current. Accuracy error can be defined as (T_2_ − T_1_)/T_1_. When the drain current is stabilized at 1.5 A, the maximum absolute difference between T_1_ and T_2_ is about 1.5 °C, corresponding to a maximum monitoring error of about 4.6%. Based on the above analysis, the integrated on-chip temperature monitoring unit has the function of the real-time monitoring of the device junction temperature, and the monitoring accuracy can reach 95%. The integrated on-chip temperature monitoring unit can monitor the junction temperature of GaN HEMT devices in real time and detect potential overheating conditions in a timely manner, thereby avoiding device damage caused by thermal stress. This real-time monitoring and early warning mechanism significantly improves the reliability and stability of the equipment. Compared with other types of measurement methods such as optical measurement, thermal resistance model prediction, and temperature-sensitive electrical parameters, this technology has significant advantages. Specifically, this approach overcomes the limitations of optical methods due to contact measurements, while avoiding the dependence on complex thermal network models and costly real-time parameter update requirements. More importantly, the method can provide junction temperature information directly and accurately, and exhibits greater flexibility and adaptability when measuring the surface temperature distribution of the device. However, over time, the sensor device itself may also age, resulting in a decrease in measurement accuracy, which requires regular calibration and maintenance of the sensor.

In practical applications, the driver chip will continuously monitor the voltage changes in the junction temperature sensing unit of the GaN HEMT device. When the detected voltage is higher than the preset threshold voltage, the built-in hysteresis comparator will flip the state and immediately output a low-level signal, thereby quickly closing the driving circuit and preventing potential damage caused by overheating. This process can quickly respond to temperature changes and ensure safe operation of the system. Once the temperature returns to normal and the voltage drops below the threshold, the comparator outputs a high level and the driver chip returns to a normal working state. This mechanism not only ensures the system’s real-time monitoring capabilities, but also greatly improves the system’s reliability and stability.

## 4. Conclusions

This work demonstrates the functionality of integrated array temperature sensors in a GaN-on-Si power HEMT, enabling real-time monitoring of chip temperature distribution under operating conditions. The on-chip array junction temperature monitoring unit was fabricated by sputtering patterned Ti/Pt thermistor strips on the surface of a GaN HEMT device. According to the test on thermal properties, the proposed junction temperature monitoring unit features a maximum temperature coefficient of resistance as high as 0.183%/°C in the range of 25 °C to 205 °C, which is comparable to that reported in the previous literature [22,23]. The test results also show that the resistance temperature coefficient values of the temperature sensors distributed at different positions are stable in the range of 0.182–0.183%°C^−1^, indicating that the prepared temperature monitoring unit exhibits excellent consistency. Additionally, the output and transfer characteristic curves of the GaN power device show that the internal integrated temperature monitoring unit has no effect on its basic electrical characteristics. Moreover, we also used a thermal imager for comparative testing to explore the accuracy of the temperature monitoring unit. The results show that the integrated temperature monitoring unit can accurately reflect the real-time temperature with a monitoring accuracy of 95%. The combination of this on-chip array junction temperature monitoring unit and the GaN HEMT device provides a novel and interesting strategy to accurately reflect the thermal distribution during device operation, which is of great significance for improving the long-term reliability of GaN power devices under high-frequency and high-power-density practical application conditions.

## Data Availability

The original contributions presented in this study are included in the article. Further inquiries can be directed to the corresponding author.
